# Recovery and Characterization of Tissue Properties from Magnetic Resonance Fingerprinting with Exchange

**DOI:** 10.3390/jimaging11050169

**Published:** 2025-05-20

**Authors:** Naren Nallapareddy, Soumya Ray

**Affiliations:** Department of Computer and Data Sciences, Case Western Reserve University, Olin 516, 2101 Martin Luther King Jr Dr, Cleveland, OH 44106, USA

**Keywords:** medical imaging, MRI, MRF, inverse problem, optimization

## Abstract

Magnetic resonance fingerprinting (MRF), a quantitative MRI technique, enables the acquisition of multiple tissue properties in a single scan. In this paper, we study a proposed extension of MRF, MRF with exchange (MRF-X), which can enable acquisition of the six tissue properties $T_{1}^{a}$,$T_{2}^{a}$, $T_{1}^{b}$, $T_{2}^{b}$, ρ and τ simultaneously. In MRF-X, ‘a’ and ‘b’ refer to distinct compartments modeled in each voxel, while ρ is the fractional volume of component ‘a’, and τ is the exchange rate of protons between the two components. To assess the feasibility of recovering these properties, we first empirically characterize a similarity metric between MRF and MRF-X reconstructed tissue property values and known reference property values for candidate signals. Our characterization indicates that such a recovery is possible, although the similarity metric surface across the candidate tissue properties is less structured for MRF-X than for MRF. We then investigate the application of different optimization techniques to recover tissue properties from noisy MRF and MRF-X data. Previous work has widely utilized template dictionary-based approaches in the context of MRF; however, such approaches are infeasible with MRF-X. Our results show that Simplicial Homology Global Optimization (SHGO), a global optimization algorithm, and Limited-memory Bryoden–Fletcher–Goldfarb–Shanno algorithm with Bounds (L-BFGS-B), a local optimization algorithm, performed comparably with direct matching in two-tissue property MRF at an SNR of 5. These optimization methods also successfully recovered five tissue properties from MRF-X data. However, with the current pulse sequence and reconstruction approach, recovering all six tissue properties remains challenging for all the methods investigated.

## 1. Introduction

Magnetic resonance imaging (MRI) is one of the most valuable tools in a clinician’s toolbox for non-invasively diagnosing soft-tissue diseases [1,2,3]. In clinical practice, a majority of MRI scans acquired are qualitative in nature. During the course of diagnosis, a radiologist might request several of these qualitative scans to obtain a complete diagnostic picture [4]. Although this approach provides valuable information to the radiologist, it restricts objective characterization of diseases and adds to patient costs and discomfort [5,6]. Additionally, variation in interpretation among radiologists could lead to significant differences in treatment plans and clinical outcomes [7].

To address some of these challenges, advanced MRI techniques such as quantitative MRI have been gaining popularity. Quantitative MRI aims to extract tissue properties such as longitudinal relaxation time $T_{1}$ (milliseconds), transverse relaxation time $T_{2}$ (milliseconds), and proton diffusion [8]. Magnetic resonance fingerprinting (MRF) is a technique that can extract multiple tissue properties from a single MRI scan [9]. Multiple tissue property images from a single scan decrease the costs and discomfort faced by patients and can improve diagnostic outcomes. Access to multiple tissue properties may also allow for the use of MRF in novel diagnostic procedures as yet unknown. However, reconstructing tissue properties from MRF images poses an algorithmic challenge due to the noisy signal generated at each voxel. The traditional approach of fitting a curve to recover tissue properties cannot be applied. Instead, the state of the art is to use a template dictionary matching approach for reconstructing tissue properties (described in detail in Section 2.2). This strategy, although robust [10], does not scale well as the number of tissue properties increases.

In this paper, we study the problem of recovering five and six tissue properties from MRF with exchange (MRF-X), where a signal from each voxel is represented by two components. We characterize and visualize the error surface formed by the inner product of an MRF-X signal and simulation of Bloch–McConnell equations derived from a range of tissue properties. We then study the use of optimization techniques to accurately retrieve tissue properties from such an MRF-X signal. Our results are promising but also reveal fundamental uncertainties about the feasibility and accuracy of acquiring multiple tissue properties. To our knowledge, no prior work has addressed the characterization or recovery problems from quantitative MRF in high dimensions.

This paper is organized as follows. In Section 2, we provide essential background on MRF and its extension, MRF-X. In Section 3, we study the error surface of MRF-X with respect to multiple tissue properties and how it changes from two to higher dimensions. In Section 4, we present a technical overview of the optimization algorithms we propose to recover tissue properties from MRF-X signals. In Section 5, we present our results applying these methods to a large dataset of simulated MRF-X signals generated with many different tissue properties and discuss the implications.

## 2. Background

### 2.1. Magnetic Resonance Fingerprinting

In standard quantitative MRI, the signal at each voxel conforms to known signal trajectories, which have well-studied mathematical models associated with them. Broadly, standard quantitative MRI uses either an exponential model of signal recovery [11,12] or a steady state signal model [13,14,15,16]. Tissue properties are then estimated by fitting acquired signal trajectories to the relevant mathematical model.

In contrast, MRF leverages recent advancements in computation power to acquire multiple tissue properties in a single scan [17]. During the acquisition of MRF, a pre-defined sequence of Radio Frequency (RF) pulses, known as the pulse sequence, is transmitted through the RF coil. This pulse sequence is governed by a set of pulse sequence parameters that control the amplitude, phase and delay of the RF pulses [18]. By carefully designing the pulse sequence, we can systematically acquire MRF signals which are sensitive to specific tissue properties. In MRF, a randomly varying acquisition scheme is employed to produce signal changes that are uniquely determined by the tissue properties present at each voxel. This variable acquisition scheme, although not unique to MRF [19], allows flexibility in pulse sequence design. Accelerated acquisition leads to corrupted MRF signal evolutions which are usually modeled as signal with added white Gaussian noise, which has shown good empirical performance [17].

### 2.2. Tissue Property Recovery from MRF Using Explicit Dictionary

Due to the complex pulse sequence employed in MRF, the signal trajectory does not follow a known closed-form function. To address this, the original MRF paper [9] proposed an explicit template dictionary-based approach. The underlying idea is to simulate known signal trajectories using an exhaustive combination of tissue property values using Bloch [20] equations.

**Figure 1:**
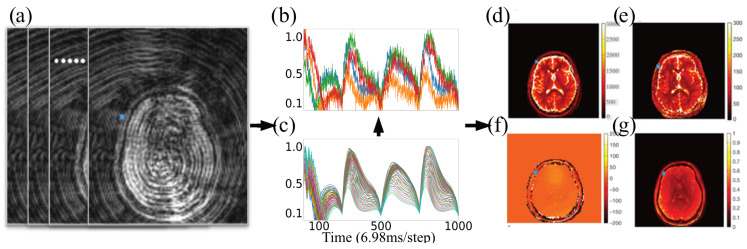
MRF tissue property mapping pipeline. Multiple noisy images (**a**) are captured with the scanner. Individual signals for each voxel (**b**) are compared with an explicit dictionary (**c**) to recover the tissue properties generating the signals. The generated maps represent tissue properties such as $T_{1}$ (**d**), $T_{2}$ (**e**), $B_{1}$ mapping (**f**), and proton density (**g**), respectively [9].

The generated signals from the Bloch simulation are collected for each combination of tissue property values into a template dictionary (Figure 1a). Next, the captured MRF signal trajectories (Figure 1b) are matched with the template dictionary to retrieve the tissue property values (Figure 1d–g) In this process, matching refers to taking an inner product between the observed signal trajectory and each element of the dictionary. The properties yielding the highest dot product (most similar known trajectory) are selected as the tissue properties. This process is repeated for each voxel to form a tissue property map. The template dictionary is a 2D matrix of values (M×N), with each column $n_{i},\;i\in \left\{1,\ldots ,N\right\}$, representing the signal evolution of a single combination of tissue properties. Each MRF acquisition strategy, such as MRF-FISP [21], uses a different number of columns (*N*) to accurately extract tissue property values. The number of columns *M* depends on several factors, including the number of tissue properties being estimated, the resolution of the tissue property values in the dictionary, and the range of tissue property values as determined by the researcher. For example, Chen et al. [22] used 20 k columns to represent the dictionary that is generated using a combination of $T_{1}$ and $T_{2}$ properties. Conversely, Hong et al. [23] employed a larger dictionary with 64 million columns to estimate four tissue properties: $T_{1}$, $T_{2}$, off-resonance, and $T_{2}^{*}$. In this context, off-resonance indicates a measure of inhomogeneity in the main magnetic field, and $T_{2}^{*}$ represents the observed or effective $T_{2}$ resulting from such inhomogeneities. As can be seen from these examples, when estimating a large number of tissue properties, explicit dictionary-based template matching can become prohibitively large.

Recent research in the field of MRF has emphasized the importance of accelerating reconstruction speed. However, it is noteworthy that many of these methods [24,25,26] continue to rely on dictionary generation. We note that generating an exhaustive dictionary with millions of entries is a computationally demanding task that can take several days to complete. Moreover, the size of the dictionary increases exponentially with the number of tissue properties being considered. Consequently, generating an explicit dictionary for more than four tissue properties can become impractical, especially in the context of clinical applications where rapid analysis is critical.

### 2.3. Magnetic Resonance Fingerprinting with Exchange (MRF-X)

In the MRF-X approach [27], a signal acquired from each voxel of the scanned region is represented by two components, labeled A and B. Each compartment is considered a distinct region that has its independent set of tissue properties ($T_{1}$, $T_{2}$) (ms). The volume ratio of compartment A is denoted by ρ. A continuous exchange of protons occurs between compartment A and compartment B. This exchange is measured using an exchange rate τ ($s^{-1}$). This modeling using two compartments has several potential uses such as the monitoring of myelin in patients with degenerative disorders of the brain and diffuse fibrosis of the heart [28]. We simulate the two-compartment model using the Bloch–McConnell equations [29], which are an extension of the Bloch equations for chemical exchange.

The acquisition of multi-component maps is constrained by certain physical limitations. In medical imaging, it is widely recognized that physical processes that occur at a rate faster than acquisition speed of the protocol cannot be measured [30]. Traditional MRI collects information at a rate that is commensurate with the $T_{1}$ and $T_{2}$ relaxation times. However, this rate is slower than the rate of chemical exchange that occurs between multiple compartments. This limitation restricts the conventional MRI’s ability to acquire chemical exchange dynamics. MRF potentially addresses this difficulty by collecting samples at a rate (TR) of 6–20 ms, which aligns with the time scale of chemical exchange between multiple components in the brain. Capitalizing on this, Hamilton et al. [27] introduced a new technique that leverages MRF to acquire multi-component maps along with chemical exchange, which they call MRF-X.

In a clinical context, multi-components maps of relaxation times with chemical exchange are not typically acquired at present. At present, the acquisition of these multi-component maps remains a challenging task due to prohibitively long scans which increase patient discomfort and associated costs. Further, validating the performance of tissue property recovery algorithms in an actual scanner will require designing phantoms with desired tissue properties so the signal from the scanner can be recorded. Currently, there are no standardized phantoms for MRF-X like the ISMRM/NIST system phantom for MRF [10]. Despite these challenges, the potential acquisition of multi-component maps may enhance diagnostic capabilities ultimately contributing to better patient outcomes.

### 2.4. MRF-X Modeling Using Bloch–McConnell Equations

The influence of chemical exchange on the magnetic resonance signal is exactly described by the Bloch–McConnell equations, which extend the standard Bloch equations to model systems with nuclei that dynamically exchange between multiple local environments. These equations incorporate the exchange rate τ that measures the transfer of magnetization accompanying the physical movement of nuclei between multiple compartments and the fractional contribution of each compartment to the overall magnetization measured using ρ. The multiple compartments have their independent relaxation properties, $T_{1}^{a}$, $T_{2}^{a}$, $T_{1}^{b}$, $T_{2}^{b}$ and are described by the following differential equation:

(1) $$\begin{array}{cc} \frac{dM}{dt} & =AM+C\\ M & =\left[\begin{array}{c} M_{x}^{A}\\ M_{y}^{A}\\ M_{z}^{A}\\ M_{x}^{B}\\ M_{y}^{B}\\ M_{z}^{B} \end{array}\right]\\ A & =\left[\begin{array}{cccccc} -\left(\frac{1}{T_{2a}}+\tau \right) & dA & 0 & \tau & 0 & 0\\ -dA & -\left(\frac{1}{T_{2a}}+\tau \right) & 0 & 0 & \tau & 0\\ 0 & 0 & -\left(\frac{1}{T_{1a}}+\tau \right) & 0 & 0 & \tau \\ \tau & 0 & 0 & -\left(\frac{1}{T_{2b}}+\tau \right) & dB & 0\\ 0 & \tau & 0 & -dB & -\left(\frac{1}{T_{2b}}+\tau \right) & 0\\ 0 & 0 & \tau & 0 & 0 & -\left(\frac{1}{T_{1b}}+\tau \right) \end{array}\right]\\ C & =\left[\begin{array}{c} 0\\ 0\\ \frac{\rho M_{\mathrm{total}}}{T_{1a}}\\ 0\\ 0\\ \frac{\left(1-\rho \right)M_{\mathrm{total}}}{T_{1b}} \end{array}\right] \end{array}$$

where *M* represents the magnetization vector, *A* is the evolution matrix, and *C* is the constant vector.

The evolution matrix *A* contains the relaxation terms ($T_{1}^{a}$, $T_{2}^{a}$, $T_{1}^{b}$, $T_{2}^{b}$), and the chemical exchange term (τ). The constant vector *C* contains the fractional contribution of each compartment to the overall magnetization measured using ρ.

The crucial connection to proton exchange described by the Bloch–McConnell equations occurs continuously and simultaneously with the evolution driven by the MRF-X pulse sequence. With each repetition time (TR) and radio frequency (RF) pulse, magnetization is constantly being redistributed between the two compartments (A and B) according to the specific exchange rate τ. This redistribution is influenced by the sequences varying RF pulses and ongoing relaxation processes. MRF probes the system’s response continuously. As a result, the exchange rate τ becomes integrated into the signal evolution captured by the MRF-X pulse sequence.

### 2.5. Deep Learning for High-Dimensional MRF

In the past decade, Artificial Intelligence (AI), and specifically Deep Learning (DL), in the context of MRI research has gained a lot of importance. In [31], the authors provide a detailed overview of the impact of AI on MRI research, from acquisition to disease prediction. Further, in the context of MRF, DL methods have been proposed as alternatives to overcome the limitations of traditional template matching.

Primarily, DL applications in MRF can be categorized into two distinct categories. The first category involves using the DL model as a faster Bloch simulator generating the dictionary elements from tissue properties for forming a template dictionary. For instance, Yang et al. [32] used a Generative Adversarial Network (GAN)-based approach to achieve a 10,000× speed increase in the generation of an MRF dictionary. Similarly, Hamilton et al. [33] used a fully connected feed-forward network to generate a cardiac MRF template dictionary, taking individual variations in heart rate and sequence timing into account.

In the second category, DL is utilized as a replacement to the complete MRF tissue property estimation pipeline, enabling generation of tissue properties from the MRF signals acquired during scanning. For example, the MRF deep reconstruction network (DRONE) proposed by Cohen et al. [34] is a four-layer deep neural network (DNN) that generates tissue properties $T_{1}$ and $T_{2}$ directly from MRF signals. The authors report that DRONE achieves results on par with conventional techniques but with a 300× speed advantage, consuming only 5% of the memory required by an explicit MRF dictionary. In other work, Fang et al. [35] leveraged spatial information of neighboring voxels in MRF, facilitating accurate quantification of tissue properties from highly undersampled MRF data. As far as we are aware, no prior work has leveraged DL to either extract multiple tissue properties or accelerate generation of explicit high-dimensional (greater than four tissue properties) MRF dictionaries. In contrast to such approaches, we focus on recovering high-dimensional tissue properties from MRF-X through optimization.

## 3. Nature of MRF Objective Function

As explained above, to find the tissue properties from a captured MRF signal, an inner product is taken between this signal and simulations from the Bloch equations. To find the true tissue properties that generated the signal, this inner product surface must be searched. The best match is the set of tissue properties that maximizes the inner product or, alternatively, minimizes an error function derived from the inner product. Thus, it is important to understand the nature of the inner product function or error function in order to understand the behavior of algorithms attempting to carry out tissue property reconstruction. In this section, we visualize the surface of the inner product function between the noisy signal, which acts as a surrogate of the data acquired from the MRI scanner, and the signals obtained from the Bloch simulations for a set of candidate tissue properties. We perform this for different tissue property dimensionalities.

Let the signal obtained from a location (i,j) on the physical surface of the scanned object be denoted X(t), where t∈{1,…,T} represents time and *T* represents the total number of time steps captured by the scanner. We denote each Bloch simulation as B(θ,t), where *t* denotes time and $\theta \in \Theta^{d}$ is the space of possible *d*-dimensional tissue properties, quantized to a suitable granularity to visualize the surface (Table 1). We will use the notation *X* and B(θ) to represent whole trajectories.

The signal captured from a scanner is typically noisy. To model this, we generate signals using additive white Gaussian noise (AWGN) with varying signal-to-noise ratios (SNRs). We denote a signal with SNR μ as $X_{\mu }$. Then, we define:

(2) $$f\left(\theta \right)=1-\frac{\langle X_{\mu },B\left(\theta \right)\rangle }{∥X_{\mu }{∥}_{2}{∥B\left(\theta \right)∥}_{2}}$$

Here, f(θ) represents the the objective (error) function surface between the input signal $X_{\mu }$ and the Bloch signal. Angular brackets 〈,〉 represent the inner product of the noisy signal from the scanner and the signal from Bloch simulation. We normalize the inner product using $l_{2}$-norm ($∥X_{\mu }{∥}_{2}$) of the noisy signal multiplied with $l_{2}$-norm (${∥B\left(\theta \right)∥}_{2}$) of the signal from Bloch simulation. We wish to find $\hat{\theta }=\arg\min_{\theta \in \Theta^{d}}f\left(\theta \right)$.

**Figure 2:**
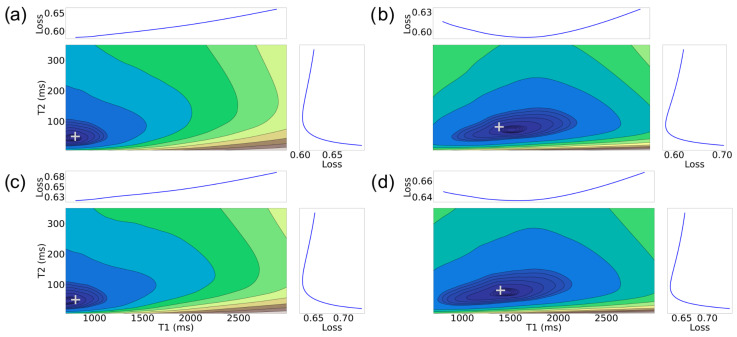
Error function f(θ) for MRF signal $X_{\mu }$ with $\theta \in \Theta^{2}=\left\{T_{1},T_{2}\right\}$. (**a**) Top-left: μ=1, [$T_{1}=800\;\mathrm{ms}$, $T_{2}=50\;\mathrm{ms}$]; (**b**) Top-right: μ=1, [$T_{1}=1400\;\mathrm{ms}$, $T_{2}=80\;\mathrm{ms}$]; (**c**) Bottom-left: μ=5, [$T_{1}=800\;\mathrm{ms}$, $T_{2}=50\;\mathrm{ms}$]; (**d**) Bottom-right: μ=5, [$T_{1}=1400\;\mathrm{ms}$, $T_{2}=80\;\mathrm{ms}$]. [$T_{1}=800\;\mathrm{ms}$, $T_{2}=50\;\mathrm{ms}$] and [$T_{1}=1400\;\mathrm{ms}$, $T_{2}=80\;\mathrm{ms}$] is indicative of white matter and gray matter in brain at 3T MRI scanner. Darker blue contours show lower *f* (better). “Loss” graphs show *f* for each axis averaged over the other axis. The symbol + indicates the true tissue property combination in each case.

In Figure 2, we show the error function for two-dimensional tissue properties, consisting of $\left\{T_{1},T_{2}\right\}$ for two specific target tissue property combinations observed in the white matter and gray matter areas of the brain, respectively [36]. We employed template matching with the explicit dictionary discussed in the Section 2.2 to generate the contour maps. This explicit dictionary consists of 10,000 elements (granularity shown in Table 1, top).

Upon examining the tissue property combination of [800 ms, 50 ms], we note that the minimum of the error surface aligns closely with the actual target, as denoted by the plus (+) symbol, for both SNR 1 and 5 cases. However, for the tissue property combination of [1400 ms, 80 ms], there is a small discrepancy between the actual tissue property and minimum of the error surface at SNR 1, indicated by the + not appearing at the center of the dark blue region. As expected, the discrepancy is reduced for the higher SNR.

From these figures, two key observations emerge. First, the sensitivity of the MRF explicit dictionary template matching procedure is dependent on the tissue property combination. This is expected from the the MRF-FISP pulse sequence’s differential sensitivity to distinct regions of the tissue property space, as highlighted in Jiang et al. [21]. Second, for low SNR, there may be a systemic mismatch that is larger than the explicit dictionary resolution. For example, Figure 2b shows that the true values lie outside the region with the lowest *f* values. This is not due to a lack of granularity of sampling based on the granularity shown in Table 1. In such a case, any algorithm relying on template matching in some form is likely to produce irreducible errors.

**Figure 3:**
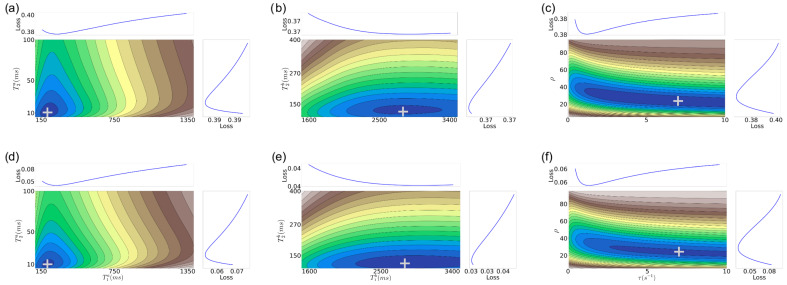
Error function f(θ) for MRF-X signal $X_{\mu }$ with $\theta \in \Theta^{6}=\left\{T_{1}^{a},T_{2}^{a},T_{1}^{b},T_{2}^{b},\tau ,\rho \right\}$. (**a**) Top-left: μ=1, [$T_{1}^{a}=200\;\mathrm{ms}$, $T_{2}^{a}=10\;\mathrm{ms}$]; (**b**) Top-middle: μ=1, [$T_{1}^{b}=2800\;\mathrm{ms}$, $T_{2}^{b}=120\;\mathrm{ms}$]; (**c**) Top-right: μ=1, [$\tau =7.0\;s^{-1}$, ρ=23.7]; (**d**) Bottom-left: μ=5, [$T_{1}^{a}=200\;\mathrm{ms}$, $T_{2}^{a}=10\;\mathrm{ms}$]; (**e**) Bottom-middle: μ=5, [$T_{1}^{b}=2800\;\mathrm{ms}$, $T_{2}^{b}=120\;\mathrm{ms}$]; (**f**) Bottom-right: μ=5, [$\tau =7.0\;s^{-1}$, ρ=23.7]. Values [$T_{1}^{a}=200\;\mathrm{ms}$, $T_{2}^{a}=10\;\mathrm{ms}$, $T_{1}^{b}=2800\;\mathrm{ms}$, $T_{2}^{b}=120\;\mathrm{ms}$, $\tau =7.0\;s^{-1}$, ρ=23.7] correspond to white matter tissue properties from [37]. Darker blue contour lines indicate lower *f* (better). “Loss” graphs show *f* along one axis averaged over the other. The “+” symbol marks the true tissue-property combination in each panel.

**Figure 4:**
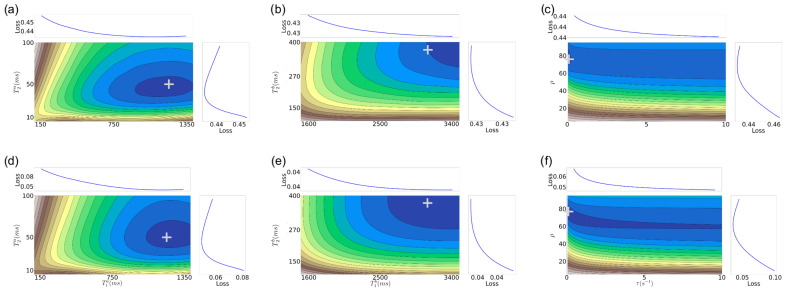
Error function f(θ) for MRF-X signal $X_{\mu }$ with $\theta \in \Theta^{6}=\left\{T_{1}^{a},T_{2}^{a},T_{1}^{b},T_{2}^{b},\tau ,\rho \right\}$. (**a**) Top-left: μ=1, [$T_{1}^{a}=1200\;\mathrm{ms}$, $T_{2}^{a}=50\;\mathrm{ms}$]; (**b**) Top-middle: μ=1, [$T_{1}^{b}=3100\;\mathrm{ms}$, $T_{2}^{b}=372\;\mathrm{ms}$]; (**c**) Top-right: μ=1, [$\tau =0.1\;s^{-1}$, ρ=76.6]; (**d**) Bottom-left: μ=5, [$T_{1}^{a}=1200\;\mathrm{ms}$, $T_{2}^{a}=50\;\mathrm{ms}$]; (**e**) Bottom-middle: μ=5, [$T_{1}^{b}=3100\;\mathrm{ms}$, $T_{2}^{b}=372\;\mathrm{ms}$]; (**f**) Bottom-right: μ=5, [$\tau =0.1\;s^{-1}$, ρ=76.6]. Values [$T_{1}^{a}=1200\;\mathrm{ms}$, $T_{2}^{a}=50\;\mathrm{ms}$, $T_{1}^{b}=3100\;\mathrm{ms}$, $T_{2}^{b}=372\;\mathrm{ms}$, $\tau =0.1\;s^{-1}$, ρ=76.6] correspond to gray matter tissue properties from [37]. Darker blue contour lines indicate lower *f* (better fits). “Loss” graphs show *f* along one axis averaged over the other. The “+” symbol marks the true tissue-property combination in each panel.

In Figure 3 and Figure 4, we show *f* for an MRF-X signal for six-dimensional tissue properties, namely ($T_{1}^{a}$,$T_{2}^{a}$,$T_{1}^{b}$,$T_{2}^{b}$,ρ,τ) for two specific target tissue property values, again motivated by values from the white and gray matter regions of the brain. Since we cannot directly visualize a 6D error surface, we consider individual 2D projections by grouping the tissue properties according to their respective compartments A and B. In such projections, we fix the other dimensions to the target values. To generate each 2D projection, we use a dictionary of 10,000 elements (granularity shown in Table 1, bottom).

From Figure 3 and Figure 4, we observe that:

- Similar to the 2D case, there are still well-defined, non-disjoint regions with minimum *f* in the space. So it is feasible (in theory) to achieve solutions close to the target values, as in the 2D case.
- The gradient structure is generally steeper in some regions than in the 2D case, as indicated by the larger number of narrower contours.
- For higher SNRs, there is still a relatively good alignment between some of the target tissue properties, such as for ($T_{1}^{a}$, $T_{2}^{a},T_{1}^{b}$, $T_{2}^{b}$), with the actual target. This observation aligns with expectations, given that the MRF-X scan is sensitive to the tissue properties $T_{1}$ and $T_{2}$ [27]. Observing ($T_{1}$, $T_{2}$) property pairs (sub-figures d,e), it is evident that the minimum of the error surface closely matches the actual target shown represented by + symbol.
- There are large “plateaus” of the error function around the minimum for some tissue properties even for high SNRs. Thus, even when the minimum aligns well with the true parameters, there may be algorithmic challenges finding it due to the error function structure, which has a combination of both steep gradients and large plateaus.
- As expected, the alignment between the minimum of the error surface and the actual target is notably more accurate for SNR 5 compared to SNR 1.
- Estimating the (ρ, τ) tissue properties is the most challenging aspect of tissue characterization. Assuming a two-compartment model, we hypothesize that the rate of exchange of protons (ρ) and the volume ratio of a compartment (without loss of generality we can assume it is the ratio of compartment b over the total volume) (τ) to the voxel are intrinsically related. If we assume that protons do not “leak” between voxels and exchange only happens inside each measured region (conservation of protons), then at equilibrium there must be an inverse relation between the rate of exchange and the proportion of the compartment to the voxel volume. This implies that multiple different combinations of tissue properties could lead to the same equilibrium state.

## 4. Methods

From our results in the previous section, it appears feasible in some cases to recover tissue properties in high dimensions by optimizing the error function, without explicitly pre-generating a template dictionary, which is infeasible in this scenario. In the following sections, we empirically evaluate several optimization algorithms to see if this can be realized in practice. First, we briefly review the methods we will evaluate below.

### 4.1. Broyden–Fletcher–Goldfarb–Shanno

Algorithm The Broyden–Fletcher–Goldfarb–Shanno (BFGS) algorithm is a popular quasi-Newtonian method used for numerical optimization. The algorithm approximates the Hessian (second derivative) matrix ($B_{k}$) of the objective function (*f*) at each iteration (*k*) to find the next iterate:

(3) $$x_{k+1}=x_{k}-\alpha_{k}B_{k}^{-1}\nabla f_{k}.$$

Here, $\nabla f_{k}$ denotes the gradient of objective function (*f*) at the current iterate ($x_{k}$), the stepsize $\alpha_{k}$ is calculated using line search such that $\alpha_{k}$ satisfies the Wolfe conditions [38], and $x_{k+1}$ is the next iterate. The algorithm is terminated when stopping criteria such as the maximum number of iterations is met. While the BFGS method can solve a wide variety of unconstrained optimization problems efficiently, a limitation for the BFGS algorithm is its lack of bound constraints. This is needed for optimizing the MRF-X objective and is solved by the extension below.

### 4.2. Limited Memory BFGS Algorithm with Bound Constraints

Limited-memory BFGS with Bound Constraints (L-BFGS-B, Algorithm 1) is a hybrid quasi-Newtonian algorithm that uses gradient projection along with a limited memory BFGS matrix update to solve large-scale nonlinear optimization problems [39]. Similar to trust region methods, L-BFGS-B approximates a quadratic at the current search point ($x_{k}$) using the approximate limited memory matrices $Y_{k}$ and $S_{k}$ composed of pairs of vectors $y_{k}$ and $s_{k}$ as follows:

(4) $$\begin{array}{cc} s_{k} & =x_{k+1}-x_{k}\\ y_{k} & =\nabla f_{k+1}-\nabla f_{k} \end{array}$$

**Algorithm 1** L-BFGS-B (*f*, $x_{0}$, ${\left[l,u\right]}^{n}$, *m*, *M*) 1:Initialize k=0, $x_{k}=x_{0}$ 2:Initialize $Y_{k},S_{k}$ to store last *m* gradient and position differences 3:**while** k≤M and $(|x_{k}-x_{k+1}|>\epsilon$ or $∥\nabla f(x_{k})∥>\epsilon )$ **do** 4:   Estimate quadratic model $\phi_{k}$ at $x_{k}$ using $Y_{k},S_{k}$ 5:   Calculate Cauchy point $x_{c}$ along projected gradient direction 6:   Determine active bounds in ${\left[l,u\right]}^{n}$ 7:   Minimize in subspace using L-BFGS Hessian approximation 8:   Update $Y_{k},S_{k}$ with new gradient and position differences 9:   k=k+110:**end while**11:**return** $x_{k},f\left(x_{k}\right)$

As a first step, the Cauchy point $x_{c}$ is estimated from a quadratic model $\phi_{k}$ of the objective function. Variables reaching their bounds are identified as the ‘active set’ and held constant, reducing the problem’s dimensionality. The algorithm then employs subspace minimization, as detailed in [39], focusing on the optimization of variables not in the active set. This subspace minimization differs from the traditional BFGS line search by maintaining variable bounds, ensuring constraints are respected while pursuing efficient optimization progress.

L-BFGS-B is widely used in practice [40] and is also appropriate for box constraints, which are essential in MRF-X. Further, unlike the BFGS algorithm, the inverse Hessian is approximated using limited memory matrices, which is computationally efficient. The computation complexity of L-BFGS-B grows linearly with number of variables, making it suitable for high-dimensional problems. However, like most gradient-based methods, it is prone to local optima. This issue is partly addressed by the approach below.

### 4.3. Simplicial Homology Global Optimization

Simplicial Homology Global Optimization (SHGO) is a global optimization algorithm designed to handle complex, high-dimensional black-box optimization problems like MRF reconstruction. The SHGO algorithm has been effectively applied to several practical applications in computed tomography [41], EEG signal extraction [42], and chemical process optimization [43]. Endres et al. [44] demonstrated competitive results against other global optimization strategies such as topographical global optimization [45] and Lc-DISIMPL [46]. Additionally, SHGO is available as part of the scipy [40,44] toolbox. These advantages have motivated us to evaluate SHGO in addressing the MRF reconstruction problem.

SHGO (Algorithm 2) begins by uniformly sampling the feasible region defined by the lower and upper bounds ${\left[l,u\right]}^{n}$ of the search space. Low-discrepancy sampling schemes such as the Sobol sequence [47] are used to decrease the probability of clusters in high-dimensional space. The number of samples *N* is a hyperparameter based on the dimensionality of the search space. The resulting set of samples P is then used as the vertices of the simplicial complex H. Triangulation (such as that by Delauney [48]) is then used to connect the edges of the vertices in the simplicial complex.
**Algorithm 2** SHGO (*f*, ${\left[l,u\right]}^{n}$, *N*, local minimizer *L*) 1:P=∅ 2:**while** |P|<N **do** 3:   X= Generate N−|P| Sobol sequence points from $R^{n}$ 4:   Scale X to bounds ${\left[l,u\right]}^{n}$ 5:   P=P∪X 6:**end while** 7:Construct simplicial complex H from f(P) 8:Generate minimizer candidates M from H; CS=∅ 9:**for** v∈M **do**10:   (x,f(x))=L(v)11:   CS{x}=(x,f(x))12:**end for**13:$x^{*}=\arg\min CS\left\{x\right\}$14:**return** $x^{*}$, $f(x^{*})$

Each vertex in the simplicial complex H consists of location, $v_{i},\;\;i\in I^{+}$, and the corresponding functional value $f(v_{i})$. The direction of each edge is evaluated based on the direction of the vector connecting two vertices on the hypersurface. For example, an edge is directed from vertex $v_{i}$ to vertex $v_{j}$ iff $f\left(v_{i}\right)<f\left(v_{j}\right)$. It can now be observed that if all the edges connected to a vertex are directed away from the vertex, the vertex forms a minimizer of the local region of the set of vertices called the star of the vertex ($st(v_{i})$). Applying Sperner’s lemma [49], there is at least a minimizer within the domain of the star of each vertex in the minimizer set M. By using the vertices in the minimizer set and a local optimization routine (L(v)) such as L-BFGS-B, the local minima can be estimated, which allows SHGO to return an approximate global minimum. The computation complexity of the SHGO algorithm without the local optimization routine is exponential in nature, which makes it infeasible for high-dimensional problems without the local optimization routine.

## 5. Results and Discussion

We now evaluate the utility of optimization algorithms to recover tissue properties from MRF-X data. The task of recovering multiple tissue properties from a single MRF-X scan poses considerable challenges. First, each tissue property varies in its sensitivity to changes in the MRF-X input signal. Second, in clinical settings, only a limited subset of the Fourier space samples are collected (undersampling). This approach inevitably leads to a tradeoff between noise and scan duration. In our study, we simulate this tradeoff by introducing Gaussian noise to the signal, mimicking the noise resulting from the undersampling performed during an actual scan. To the best of our knowledge, our study is the first of its kind to explore the characterization of six-tissue-property MRF (termed as MRF-X) recovery using various nonlinear optimization techniques.

To simulate MRF-X data, we employed a modified version of an MRF-FISP pulse sequence, as described in [21]. Specifically, the MRF-X sequence uses a variable flip angle between 0 and $60^{\circ }$ and maintains a constant repetition time (TR) of 6.98 ms. As explained in [37], an inversion pulse is introduced before specific RF excitations at the setting inversion time (TI) [21 ms, 100 ms, 250 ms]. This allows the pulse sequence to be more sensitive to $T_{1}$ [50]. We set the SNR μ=5.0 for these data.

For the implementation, we built a Bloch–McConnell simulator in C++ using the Eigen linear algebra library [51]. All optimization routines were written in Python 3.8 using numpy and scipy. The simulations were run on a server with 2×20-core Intel Xeon CPUs and 384 GB total system memory, custom-built by Puget Systems, Inc. (Auburn, WA, USA). The simulation took ∼1 min to generate a single signal with a given tissue property combination. Code for the simulation and parameter recovery will be made available on GitHub (https://github.com/).

### 5.1. MRF Results

**Figure 5:**
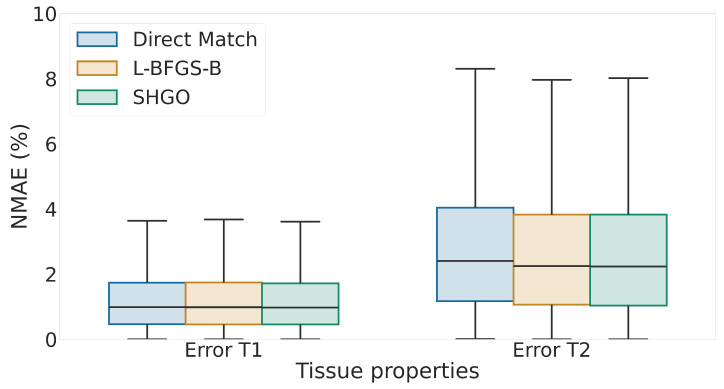
Box-and-whisker plot of normalized mean absolute error as percentage for tissue properties $T_{1}$ and $T_{2}$. We compare the algorithms direct matching using explicit dictionary with L-BFGS-B and SHGO with L-BFGS-B.

We start by validating L-BFGS-B and SHGO with L-BFGS-B as the local minimizer (SHGO+L-BFGS-B), for tissue properties $T_{1}$ and $T_{2}$ against the standard explicit dictionary template matching technique [9]. To create this dictionary, we generate 10,000 data points from a combination of the properties $T_{1}$ and $T_{2}$. In this dictionary, the $T_{1}$ values range from 500 ms to 3000 ms, while the $T_{2}$ values range from 20 ms to 350 ms. For validation, target signals are generated by randomly sampling values multiple times within the same range as the dictionary. To emulate realistic signals obtained at the scanner, we introduce white Gaussian noise with an SNR of 5 into the target signals. Each optimization algorithm then produces estimated ($T_{1}$, $T_{2}$) values, from which we compute the normalized mean absolute difference (NMAE) as an absolute error relative to the input properties. The results are shown in Figure 5.

From Figure 5, we observe that all methods produce comparable results in this scenario. In particular, the dictionary-free optimization approaches produce excellent results, with error typically in the 2–4% range, in line with direct matching. We also observe that the error in the $T_{2}$ tissue property is typically worse than the error in $T_{1}$; this is due to the sensitivity difference to the MRF signal between different tissue properties. This experiment shows that optimization approaches can recover signals commensurate with dictionary matching without generating a complete dictionary from MRF signals.

### 5.2. MRF-X Results

In our experiments, we analyze MRF-X using two distinct scenarios: one with five tissue properties (5D) and the other with six (6D). In the 5D scenario, we set the tissue property τ to be 0. This setting corresponds to a two-compartment model of the tissue, where there is no movement of protons between the compartments. We adopt this approach to highlight the effect of the tissue property τ in estimation of the tissue property ρ and to explore the impact of dimensionality on our optimization procedures. To evaluate the methods, we create two datasets: one consisting of 24,000 target properties in 6D and another consisting of 20,000 properties in 5D as follows. We first sample 1000 points using a regular grid sampling scheme across the tissue property ranges given in Table 2 (left). Then, for each sampled point, we fix all but one of the properties and resample the remaining properties three more times evenly across their ranges. This ensures that each axis is being well sampled, in a tractable manner, for many different values of the other properties. It is similar to Latin hypercube sampling, though modified to use a fixed total sample while still maximizing the sampling of the full hyperspace. We therefore expect our results to generalize well across the whole 5D or 6D spaces.

In the case of MRF-X, generating an explicit dictionary with the necessary resolution to effectively extract tissue properties becomes intractable. However, we use a modified dictionary-based matching approach that we term **Gold Standard** to establish a performance bound for optimization methods.

The Gold Standard dictionary is designed by restricting the extent of tissue properties used in the dictionary to a constrained region around the actual tissue property value (which would be unknown in reality) in a six-dimensional space. The specifics of each tissue property’s extent are detailed in Table 2 (right). To generate a dictionary (separately for each possible target property combination we test), we randomly sample 1000 points within this space. We observe from the “Max error” column of the table that this sampling introduces limited error in the estimates of the individual tissue properties. It is important to emphasize that this approach is not a baseline in that it could not be performed in practice; however, it gives us an idea of the irreducible error (and so an upper bound on the best performance) in this space. That is why we label it “**Gold Standard**”.

In Table 3 and Table 4, we show tissue property recovery errors for 5D and 6D for two optimization methods: *L-BFGS-B* and *SHGO* combined with *L-BFGS-B* for local search. These methods are initialized randomly from the hypercube defined by the tissue property bounds in Table 2. We also show results for two Gold Standard derivatives: explicit dictionary matching with the Gold Standard dictionary (“Dictionary matching w/Gold Std.”) and *L-BFGS-B* with Gold Standard initialization (“L-BFGS-B w/Gold Std.”). This method runs *L-BFGS-B* algorithm, initialized randomly within a hypercube determined by the edge length from the ’Extent’ column in Table 2 centered around the true tissue property values. Since the two latter methods use the knowledge of the true tissue properties, they serve as upper bounds on potential performance. Our results are shown in terms of NMAE along with standard deviation across the 20,000 points (in 5D) or 24,000 points (in 6D).

Further, we conducted a timing comparison of SHGO and SHGO+L-BFGS-B for the 6D case. In this comparison, we did not use the “Gold Standard” dictionary matching as it is infeasible in practice. For the L-BFGS-B algorithm, our results show that it takes on average 41 s to recover a single tissue property with a standard deviation of 12 s on a 16 core AMD CPU with 32 GB of memory. On the other hand, SHGO+L-BFGS-B takes on average 190 s with a standard deviation of 48 s on a 16 core AMD CPU with 32 GB of memory. This indicates that SHGO+L-BFGS-B is relatively slower than L-BFGS-B for the recovery problem but not as significantly slower as suggested by exponential scaling of the SHGO algorithm. By using the L-BFGS-B as the local minimizer, we significantly offset the computational cost of the SHGO algorithm.

Looking at the five tissue property results, we observe that (i) there is an irreducible error of 2–6% in each tissue property. This error is present even if we use explicit matching within a small radius of the true tissue property. Thus, we cannot expect practical methods to achieve lower error than this on average across the space of 5D properties. (ii) The error rates of *L-BFGS-B w/Gold Std.* and *L-BFGS-B* are comparable in most cases, except for ρ. This indicates that on average, the *L-BFGS-B* method (initialized randomly) is able to get close to the “Gold Standard” hypercube. However, within the “Gold Standard” hypercube the local gradient may not be smooth and the minimum may not be at the zero of the gradient. This is also illustrated in Figure 3 and Figure 4 for the 6D case and is likely the reason why the *Dictionary match w/Gold Std.* method produces an irreducible error. (iii) We observe that *SHGO + L-BFGS-B* generally produces better results than *L-BFGS-B* on its own. This indicates that this global optimization approach is able to get closer to the “Gold Standard” hypercube than *L-BFGS-B* and pick better minimizer candidates on average. However, since *L-BFGS-B* is the local minimizer, the final error is not less than *L-BFGS-B w/Gold Std*. It is a direction for future work to evaluate if a different minimizer using other signals than just the local gradient could work better with SHGO for this problem. (iv) The ρ tissue property is the hardest to estimate accurately using the local gradient alone. However, given that *Dictionary match w/Gold Std.* achieves an average error rate of 4.5%, it seems likely there are other features beyond the local gradient that could be exploited to find better solutions.

In the six-tissue-property case, again, there is an irreducible error of 2–9% in each tissue property on average. Perhaps surprisingly, *L-BFGS-B w/Gold Std.* is able to produce better results on average than in the five-tissue-property case for $T_{1}^{a}$, $T_{1}^{b}$, and $T_{2}^{a}$. Between *L-BFGS-B* and *SHGO + L-BFGS-B*, the latter is once again the better-performing method. It approaches the results of *L-BFGS-B w/Gold Std.*, though not surprisingly, the error rates are higher than in the 5D case. It is interesting that the error rates for *SHGO + L-BFGS-B*, though higher, are not *substantially* higher (usually less than 5%) other than $T_{2}^{b}$ in the 5D case given the substantially larger space being explored. Finally, we observe that the ρ and τ properties produce high errors when estimated by *L-BFGS-B* and *SHGO + L-BFGS-B*. Since the error is lower for the “Gold Standard” methods, it seems likely that the methods are being misdirected into regions where the true property is not present. As we discussed in Section 3, these two properties may not be independent, so there may be multiple pairs of (ρ, τ) solutions that yield similar behavior. To support this claim, we observe that the ρ estimates form both “Gold Standard” methods have either a high error or a wide confidence interval, and the τ estimates have high irreducible error and large confidence intervals. This indicates it may be inherently difficult to estimate these properties jointly.

## 6. Conclusions

In this paper, we present a systematic analysis of tissue-property recovery from MRF-X signals. We first visualize the surface of the error function that is explored to recover tissue properties in 2D and 6D cases. The visualization illustrates how the gradient signal changes from 2D to 6D for different target tissue properties and highlights the fact that there is structure that can be exploited but also difficulties caused by plateaus and lack of alignment of the minimums with the target properties at lower SNRs.

Based on this analysis, we show results for two optimization algorithms, *L-BFGS-B* and *SHGO + L-BFGS-B* on 5D and 6D tissue property-recovery problems, as well as two “Gold Standard” methods the illustrate the best that can be achieved. In the 5D case, *SHGO + L-BFGS-B* outperforms *L-BFGS-B* and comes within 10-20% of the error values achieved by *L-BFGS-B* with “Gold Standard” initialization. In the 6D case, *SHGO + L-BFGS-B* again outperforms *L-BFGS-B* for most tissue properties. The errors are comparable to the 5D case for the three tissue properties. However, ρ and τ seem to be hard to estimate in combination, even for the “Gold Standard” approaches.

Key takeaways:

- Estimating six tissue properties (especially ρ and τ) from MRF-X signals is a challenging optimization problem due to complex error surfaces with plateaus and misaligned minima at lower SNRs.
- Because of the lack of standard optimization approaches for this problem, we created a “Gold Standard” which, although impractical in clinical practice, provides a baseline for comparison of our results.
- Our proposed SHGO + L-BFGS-B algorithm comes within 10–20% of L-BFGS-B with “Gold Standard” initialization, demonstrating its effectiveness for practical applications.
- The 6D recovery problem (including both ρ and τ) presents fundamental challenges, with these two parameters being particularly difficult to estimate simultaneously.
- Visualization of the error surfaces reveals an exploitable structure that can guide the development of more effective optimization strategies for MRF-X tissue property recovery.

In future work, we plan to investigate the use of alternative local optimization strategies with SHGO that can take advantage of more than local gradient information, as well as techniques to better estimate the τ and ρ properties in 6D.

To summarize, our study provides an analysis and shows results and potential challenges in recovering multiple tissue properties from a single MRI scan. While simultaneously estimating multiple tissue properties poses considerable technical and fundamental physics challenges, we believe this work is an important step towards developing robust tools for quantitative multi-parametric MRI, advancing its potential as a powerful diagnostic tool in clinical practice.

## Figures and Tables

**Table 1:** Ranges and granularities of tissue property values in 2D and 6D models.

Tissue Property	Min	Max	Step
Two-tissue property model			
$T_{1}$ (ms)	700	3000	0.23
$T_{2}$ (ms)	5	350	0.03
Six-tissue property model			
$T_{1}^{a}$ (ms)	100	1400	13
$T_{2}^{a}$ (ms)	5	100	0.95
$T_{1}^{b}$ (ms)	1500	3500	20
$T_{2}^{b}$ (ms)	100	400	3
τ ($s^{-1}$)	0.05	10	0.01
ρ (%)	5	95	0.9

**Table 2:** Boundary constraints (minimum and maximum) for L-BFGS-B and SHGO algorithms, along with Gold Standard dictionary’s overall range (“Extent”) and the maximum possible error. Here, “Extent” denotes the difference between the minimum and maximum values, with actual tissue properties centered within this interval.

	Boundaries		Gold Std.	
Property	Minimum	Maximum	Extent	Max Error.
$T_{1}^{a}$ (ms)	800	1400	300	0.18
$T_{2}^{a}$ (ms)	20	150	20	0.50
$T_{1}^{b}$ (ms)	1500	2800	300	0.10
$T_{2}^{b}$ (ms)	200	350	20	0.05
τ ($s^{-1}$)	0.1	5	0.3	1.5
ρ (%)	5	95	6	0.6

**Table 3:** Normalized mean absolute errors with standard deviation for five tissue properties. Values are presented as mean±standarddeviation.

TP	Dictionary Match with Gold Std. *	L-BFGS-B with Gold Std. *	L-BFGS-B	SHGO + L-BFGS-B
$T_{1}^{a}$	0.059±0.042	0.123±0.128	0.137±0.136	0.126±0.128
$T_{2}^{a}$	0.053±0.056	0.228±0.451	0.276±0.588	0.249±0.457
$T_{1}^{b}$	0.024±0.013	0.105±0.124	0.124±0.133	0.126±0.130
$T_{2}^{b}$	0.058±0.062	0.149±0.175	0.150±0.163	0.148±0.168
ρ	0.045±0.046	0.361±0.772	0.563±1.413	0.420±0.830

[*] Gold Standard (“Gold Std.”) denotes template matching within a region constrained around the solution. L-BFGS-B with Gold Standard (“LBFGS w/Gold Std.”) refers to the L-BFGS-B algorithm with initial guess derived from region constrained around the solution. SHGO with L-BFGS-B (“SHGO+L-BFGS-B”) is the global optimization SHGO using L-BFGS-B as its local minimizer. Better L-BFGS-B and SHGO+L-BFGS-B error rates are highlighted using bold font.

**Table 4:** Normalized mean absolute errors with standard deviation for six tissue properties. Values are presented as mean±standarddeviation.

TP	Dictionary Match with Gold Std. *	L-BFGS-B with Gold Std. *	L-BFGS-B	SHGO + L-BFGS-B
$T_{1}^{a}$	0.040±0.041	0.076±0.101	0.159±0.124	0.137±0.120
$T_{2}^{a}$	0.038±0.048	0.202±0.310	0.342±0.457	0.284±0.337
$T_{1}^{b}$	0.034±0.026	0.066±0.098	0.187±0.155	0.169±0.133
$T_{2}^{b}$	0.023±0.018	0.186±0.274	0.293±0.267	0.278±0.227
ρ	0.092±0.144	0.028±1.040	1.720±1.413	3.860±3.930
τ	0.424±0.830	0.245±0.815	1.870±3.360	1.530±4.420

[*] Gold standard (“Gold Std.”) denotes template matching within a region constrained around the solution. L-BFGS-B with Gold Standard (“LBFGS w/Gold Std.”) refers to the L-BFGS-B algorithm with initial guess derived from region constrained around the solution. SHGO with L-BFGS-B (“SHGO+L-BFGS-B”) is the global optimization SHGO using L-BFGS-B as its local minimizer. Better L-BFGS-B and SHGO+L-BFGS-B error rates are highlighted using bold font.

## Data Availability

Code and data will be made available on github upon publication.
